# Treatment of Focal-Onset Seizures in Children: Should This Be More Etiology-Driven?

**DOI:** 10.3389/fneur.2022.842276

**Published:** 2022-03-07

**Authors:** Alec Aeby, Berten Ceulemans, Lieven Lagae

**Affiliations:** ^1^Pediatric Neurology, Queen Fabiola Children's University Hospital, Université Libre de Bruxelles (ULB), Brussels, Belgium; ^2^Department of Pediatric Neurology, Antwerp University Hospital, University of Antwerp, Antwerp, Belgium; ^3^Reference Center for Refractory Epilepsy, Pediatric Neurology, Department of Development and Regeneration, University Hospitals Leuven, Leuven, Belgium

**Keywords:** focal-onset seizure, epilepsy, children, antiseizure medication, syndrome, etiology

## Abstract

To accelerate the process of licensing antiseizure medication (ASM) in children, extrapolation of efficacy data for focal-onset seizures from adults to children ≥2 or ≥4 years of age is now accepted. We summarized the efficacy evidence from randomized, controlled trials that was used to grant approval for the pediatric indication of focal-onset seizures for the different ASMs available in Europe. Data from high-quality randomized, controlled trials in young children are limited, especially on the use of ASMs in monotherapy. Licensure trials are typically focused on seizure type irrespective of etiology or epilepsy syndrome. We elaborate on the importance of etiology- or syndrome-driven research and treatment, illustrating this with examples of childhood epilepsy syndromes characterized by predominantly focal-onset seizures. Some of these syndromes respond well to standard ASMs used for focal-onset seizures, but others would benefit from a more etiology- or syndrome-driven approach. Advances in molecular genetics and neuroimaging have made it possible to reveal the underlying cause of a child's epilepsy and tailor research and treatment. More high-quality randomized, controlled trials based on etiology or syndrome type are needed, including those assessing effects on cognition and behavior. In addition, study designs such as “N-of-1 trials” could elucidate possible new treatment options in rare epilepsies. Broadening incentives currently in place to stimulate the development and marketing of drugs for rare diseases (applicable to some epilepsy syndromes) to more common pediatric epilepsy types and syndromes might be a means to enable high-quality trials, and ultimately allow more evidence-based treatment in children.

## Introduction

Epilepsy, a chronic neurological disorder characterized by the recurrence of unprovoked seizures, affects nearly 50 million people worldwide (1). The epilepsy prevalence is highest in young children and older adults (1–3) and was estimated to be 6.2/1,000 people globally in 2016 (1). Epileptic seizures can initiate in one region of one hemisphere of the brain—focal-onset seizures—or in both hemispheres—generalized-onset seizures (4). Epilepsy types and syndromes can be characterized by either exclusively focal or generalized seizures or by a combination of both (5).

Causes of epilepsy include genetic mutations, infections, metabolic disorders, immune disorders and structural abnormalities (5). Determining the etiology of epilepsy may allow optimizing treatment; for instance, certain focal brain malformations can be amenable to curative surgery, and the identification of a genetic mutation may enable effective targeted treatment (3, 6).

The diagnosis and management of epilepsy in children pose unique challenges. Misdiagnosis is common, as paroxysmal events are often confused with epilepsy in children (7), and, in neonates, diagnosis is often impossible without continuous electroencephalography monitoring (8, 9). Seizures in infancy and childhood can have detrimental effects on behavior and cognition, and early, effective treatment is therefore essential (3, 6, 10, 11). However, antiseizure medication (ASM) can itself negatively affect neurodevelopment (6, 11, 12). Benefits and side effects should therefore be carefully weighed when choosing ASMs for children.

Because of the methodological and ethical challenges associated with randomized, placebo-controlled trials in children, ASMs are typically first tested and licensed in adults, and it may take years before a new drug is approved in children and infants (6, 13–16). Off-label ASM use in children is therefore common (17–20). Recognizing that this poses risks to treated children and may further complicate the conduct of placebo-controlled trials and delay pediatric drug approval, regulators now accept extrapolation of efficacy data for focal-onset seizures from adults to children ≥2 or ≥4 years old because the pathophysiology of focal-onset seizures and their responsiveness to ASMs are considered largely comparable in both populations (13, 15, 21, 22). However, as the etiology of epilepsy in adults differs from that in children, this extrapolation might be too simplistic, and some experts advocate etiology-driven treatment decisions. Tolerability and safety cannot be extrapolated and should be evaluated in prospective studies that may be single-arm and open-label (13, 15, 21, 22). Effects on behavior and cognition are particularly important to assess, but data are still limited (6, 12). Furthermore, dosing regimens cannot be extrapolated to children (particularly newborns and young infants) because pharmacokinetic properties of drugs (absorption, drug distribution, metabolism, and excretion) evolve with age due to physiological and anatomical changes (23).

In this review, we summarize the efficacy evidence that was used to approve the pediatric indication (patients <18 years) of focal-onset seizures for the ASMs available in Europe and highlight challenges related to the extrapolation of adult data to children. We also elaborate on the importance of etiology- and syndrome-driven research and treatment decisions, using examples of childhood epilepsy syndromes characterized by predominantly focal-onset seizures.

## Pharmacological Treatment of Focal-Onset Seizures in Children and Adolescents

### ASMs for Focal-Onset Seizures in Children and Adolescents

**Figure 1:**
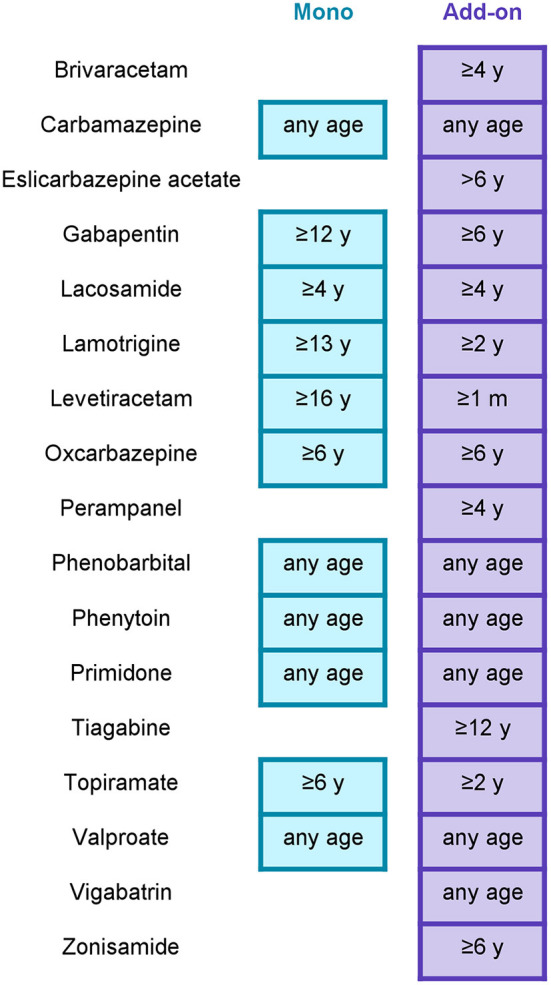
Antiseizure medication approved in Europe for focal-onset seizures in children and adolescents. “Any age” includes neonatal use. Mono, approved for monotherapy; add-on, approved for adjunctive therapy; m, months of age; y, years of age. This figure only pertains to focal-onset seizures in children; some of these drugs are also indicated for the treatment of other seizure types and/or of focal-onset seizures in adults. Indications were taken from the summaries of product characteristics available at: https://www.ema.europa.eu/en/medicines, https://mri.cts-mrp.eu/Human/Product/FullTextSearch, or http://www.hpra.ie/homepage/medicines/medicines-information/find-a-medicine/ depending on whether the drug was authorized through a centralized procedure or at national level. In addition to the listed drugs, a small number of drugs have been approved in Europe for use in specific childhood epilepsy syndromes accompanied by focal-onset seizures (e.g., sulthiame for childhood epilepsy with centrotemporal spikes, everolimus for tuberous sclerosis complex, cannabidiol for tuberous sclerosis complex and Dravet syndrome, fenfluramine for Dravet syndrome).

In Europe, 17 ASMs are indicated for the adjunctive treatment of focal-onset seizures in children and/or adolescents (Table 1, Figure 1). Of these, 11 are also approved as monotherapy. Indications differ in terms of the lower age limit at which treatment can be given, with only the first-generation ASMs (carbamazepine, phenobarbital, phenytoin, primidone, and valproate), vigabatrin and levetiracetam indicated to treat focal-onset seizures in infants (<1 year; the last two only as adjunctive treatment). For second-generation ASMs (gabapentin, lamotrigine, levetiracetam, oxcarbazepine, tiagabine, topiramate, vigabatrin, and zonisamide), licensure in children or adolescents was based on randomized, controlled trials in the pediatric population (Table 1) (66–75). The four most recent drug approvals for focal-onset seizures in children <12 years old (i.e., for the third-generation ASMs eslicarbazepine acetate, lacosamide, brivaracetam, and perampanel) were granted by the European Medicines Agency (EMA) based on extrapolation of adult or adolescent efficacy data (Table 1) (76–79).

**Table 1:** Antiseizure medication with a pediatric indication for focal-onset seizures in Europe and summary of efficacy evidence from randomized, placebo-controlled trials^a^ used as basis for these indications.

**References**	**Mono or add-on**	**Age**	**N**	**Etiology^**b**^**	**Efficacy (primary outcome)^**c**^**
**Brivaracetam (BRV)—pediatric FOS indication: add-on**, **≥4 years (extrapolation)**					
Biton et al. (24)	Add-on	16–70 years	400	NA	*% reduction* vs. *PBO in weekly FOS frequency:* BRV 5 mg:−0.9%, ns; BRV 20 mg: 4.1%, ns; BRV 50 mg: 12.8%, s
Ryvlin et al. (25)	Add-on	16–70 years	399	NA	*% reduction* vs. *PBO in weekly FOS frequency:* BRV 20 mg: 6.8%, ns; BRV 50 mg: 6.5%, ns; BRV 100 mg: 11.7%, s
Klein et al. (26)	Add-on	16–80 years	768	NA	*≥50% FOS responder rate:* PBO: 21.6%; BRV 100 mg: 38.9%, s; BRV 200 mg: 37.8%, s
**Carbamazepine (CBZ)—pediatric FOS indication: mono and add-on, any age**					
No randomized, PBO-controlled trials at the time of licensure					
**Eslicarbazepine acetate (ESL)—pediatric FOS indication: add-on**, **>6 years (extrapolation)**					
Elger et al. (27)	Add-on	≥18 years	402	NA	*LSM log difference* vs. *PBO in 4-week seizure frequency:* ESL 400 mg: −0.08, ns; ESL 800 mg: −0.19, s; ESL 1,200 mg: −0.22, s
Gil-Nagel et al. (28)	Add-on	≥18 years	252	NA	*LSM difference* vs. *PBO in 4-week seizure frequency:* ESL 800 mg: −1.6, s; ESL 1,200 mg: −1.9, s
Ben-Menachem et al. (29)	Add-on	≥18 years	395	NA	*LSM difference* vs. *PBO in 4-week seizure frequency:* ESL 400 mg: −1.5, ns; ESL 800 mg: −3.8, s; ESL 1,200 mg: −3.5, s
Sperling et al. (30)	Add-on	≥16 years	653	NA	*LSM log difference* vs. *PBO in 4-week seizure frequency:* ESL 800 mg: −0.18, ns; ESL 1,200 mg: −0.26, s
Kirkham et al. (31)	Add-on	2–18 years	304	Congenital/hereditary disorder: 20.9% Idiopathic: 14.2% Infectious disease: 9.7% Cranial trauma/injury: 6.0% Cerebrovascular disease: 6.0% Brain tumor: 2.2% Systemic/toxic/metabolic disorder: 0.7% Unknown: 24.6% Other: 20.9%	≥*50% responder rate*: PBO: 31.0%; ESL 20 mg/kg: 30.6%, ns
Józwiak et al. (32)	Add-on	6–16 years	123	NA	*≥50% responder rate:^*d*^* PBO: 25.0%; ESL 30 mg/kg: 50.6%, s
**Gabapentin (GBP)—pediatric FOS indication: mono**, **≥12 years; add-on**, **≥6 years**					
UK Gabapentin Study Group (33)^e^	Add-on	14–73 years	127	NA	*≥50% FOS responder rate:* PBO: 9%; GBP 1,200 mg: 23%, s
The US Gabapentin Study Group (34)^e^	Add-on	≥16 years	306	NA	*≥50% FOS responder rate:* PBO: 8.4%; GBP 600 mg: 18.4%, ns; GBP 1,200 mg: 17.6%, ns; GBP 1,800 mg: 26.4%, s
Anhut et al. (35)^e^	Add-on	≥12 years	272	NA	*Median % reduction in FOS frequency* vs. *baseline:* PBO: 0.3%; GBP 900 mg: 21.8%; GBP 1,200 mg: 17.8%
Appleton et al. (36)	Add-on	3–12 years	247	NA	*LSM of response ratio for FOS:* PBO: −0.072; GBP 23–35 mg/kg: −0.161, s
**Lacosamide (LCM)—pediatric FOS indication: mono and add-on**, **≥4 years (extrapolation, confirmed by pediatric randomized add-on trial)**					
Ben-Menachem et al. (37)	Add-on	18–65 years	421	NA	*≥50% responder rate:* PBO: 21.9%; LCM 200 mg: 32.7%, ns; LCM 400 mg: 41.1%, s; LCM 600 mg: 38.1%, s
Halász et al. (38)	Add-on	16–70 years	485	NA	*≥50% FOS responder rate:* PBO: 25.8%; LCM 200 mg: 35.0%, ns; LCM 400 mg: 40.5%, s
Chung et al. (39)	Add-on	16–70 years	405	NA	*≥50% FOS responder rate:* PBO: 18.3%; LCM 400 mg: 38.3%, s; LCM 600 mg: 41.2%, s
Farkas et al. (40)	Add-on	4–16 years	343	NA	*Median % reduction in FOS frequency* vs. *baseline:* PBO: 21.7%; LCM 6–12 mg/kg: 51.7%
Baulac et al. (41)^f^	Mono	≥16 years	888	NA	*Kaplan-Meier % of patients 6 months seizure free (last assessed dose):* CBZ-CR 400–1,200 mg: 91.1%; LCM 200–600 mg: 89.8%, non-inferiority met
**Lamotrigine (LTG)—pediatric FOS indication: mono** **≥13 years; add-on**, **≥2 years**					
Duchowny et al. (42)^e^	Add-on	2–16 years	199	Idiopathic: 38% Symptomatic: 62%	*Median % reduction in FOS frequency* vs. *baseline:* PBO: 6.7%; LTG 1–15 mg/kg: 36.1%, s
Piña-Garza et al. (43)	Add-on	1–24 months	38	NA	*% treatment failure*: PBO: 84%; LTG 5.1–15.6 mg/kg: 58%, ns
**Levetiracetam (LEV)—pediatric FOS indication: mono** **≥16 years; add-on**, **≥1 month**					
Glauser et al. (44)	Add-on	4–16 years	216	NA	*% reduction* vs. *PBO in weekly FOS frequency:* LEV 60 mg/kg: 26.8%, s
Piña-Garza et al. (45)	Add-on	1 month−3 years	116	Unknown etiology: 26.7% Genetic origin (familial epilepsy): 5.0% Congenital malformation: 26.7% Perinatal events: 30.0% Cranial trauma: 3.3% Brain surgery: 0.0% Primary degenerative lesion: 1.7% Cerebrovascular accident: 3.3% Cerebral infection: 6.7% Other: 10.0%	*≥50% FOS responder rate:* PBO: 19.6%; LEV 40–50 mg/kg: 43.1%, s
Brodie et al. (46)^f^	Mono	≥16 years	579	NA	*% of patients 6 months seizure free (last assessed dose):* CBZ-CR 400–1,200 mg: 72.8%; LEV 1,000–3,000 mg: 73.0%, non-inferiority met
**Oxcarbazepine (OXC)—pediatric FOS indication: mono and add-on**, **≥6 years**					
Barcs et al. (47)^e^	Add-on	15–65 years	694	NA	*Median % reduction in seizure frequency* vs. *baseline:* PBO: 7.6%; OXC 600 mg: 26.5%, s; OXC 1,200 mg: 40.2%, s; OXC 2,400 mg: 50.0%, s
Glauser et al. (48)^e^	Add-on	3–17 years	267	NA	*Median % reduction in FOS frequency* vs. *baselin*e: PBO: 9%; OXC 30–46 mg/kg: 35%, s
Piña-Garza et al. (49)^e^, ^g^	Add-on	1 month−3 years	128	NA	*Median absolute reduction in type 1 seizure frequency:* OXC 10 mg/kg: 1.4; OXC 60 mg/kg: 2.0, s
Schachter et al. (50)^e^	Mono	11–65 years	102	NA	*Time to exit:* Significantly in favor of OXC 2,400 mg vs. PBO
Sachdeo et al. (51)^e^, ^g^	Mono	≥12 years	96	NA	*Time to exit:* Significantly in favor of OXC 2,400 mg vs. OXC 300 mg
Beydoun et al. (52)^e^, ^g^	Mono	≥12 years	87	NA	*Exit rate:* OXC 300 mg: 93.3%; OXC 2,400 mg: 41.2%, s
**Perampanel (PER)—pediatric FOS indication: add-on**, **≥4 years (extrapolation)**					
French et al. (53)	Add-on	≥12 years	390	NA	*≥50% responder rate*: PBO: 26.4%; PER 8 mg: 37.6%, ns; PER 12 mg: 36.1%, ns
French et al. (54)	Add-on	≥12 years	389	NA	*≥50% responder rate:* PBO: 14.7%; PER 8 mg: 33.3%, s; PER 12 mg: 33.9%, s
Krauss et al. (55)	Add-on	≥12 years	712	NA	*≥50% responder rate*: PBO: 17.9%; PER 2 mg: 20.6%, ns; PER 4 mg: 28.5%, s; PER 8 mg: 34.9%, s
Rosenfeld et al. (56) [pooled analysis of (53–55)]	Add-on	12–17 years	143	NA	*≥50% responder rate:* PBO: 22.2%; PER 2 mg: 4.8%; PER 4 mg: 23.1%; PER 8 mg: 40.9%; PER 12 mg: 45.0%
**Phenobarbital (PB)—pediatric FOS indication: mono and add-on, all ages**					
No randomized, PBO-controlled trials at the time of licensure					
**Phenytoin (PHT)**—**pediatric FOS indication: mono and add-on, all ages**					
No randomized, PBO-controlled trials at the time of licensure					
**Primidone (PRI)—pediatric FOS indication: mono and add-on, all ages**					
No randomized, PBO-controlled trials at the time of licensure					
**Tiagabine (TGB)—pediatric FOS indication: add-on**, **≥12 years**					
Uthman et al. (57)^e^	Add-on	12–77 years	297	NA	*Median % reduction in impaired aware FOS frequency* vs. *baseline*: PBO: 11%; TGB 16 mg: 13%, ns; TGB 32 mg: 25%, s; TGB 56 mg: 33%, s
Sachdeo et al. (58)^e^	Add-on	12–75 years	318	Idiopathic: 52% Trauma: 28% Infection: 21% Genetic propensity: 18% Antenatal/perinatal injury: 16%	*Median absolute reduction in impaired aware FOS frequency* vs*. baseline*: TGB 2 × 16 mg: 1.6, ns; TGB 4 × 8 mg: 1.2, s
**Topiramate (TPM)—pediatric FOS indication: mono**, **≥6 years; add-on** **≥2 years**					
Elterman et al. (59)	Add-on	2–16 years	86	NA	*% reduction in FOS frequency* vs. *baseline*: PBO: 10.5%; TPM 6 mg/kg: 33.1%, s
Arroyo et al., Glauser et al. (60, 61)^g^	Mono	6–15 years (subgroup analysis)	151^h^	NA	*Time to first seizure:* Significantly longer with TPM 400 mg than TPM 50 mg
Privitera et al., Wheless et al. (62, 63)^i^	Mono	6–16 years (subgroup analysis)	119^h^	NA	*Time to exit*: No significant difference between TPM 100 mg, TPM 200 mg and CBZ 600 mg or VPA 1,250 mg
**Valproate (VPA)—pediatric FOS indication: mono and add-on, all ages**					
No randomized, PBO-controlled trials at the time of licensure					
**References**	**Mono or add-on**	**Age**	**N**	**Etiology^**b**^**	**Efficacy (primary outcome)^**c**^**
**Vigabatrin (VGB)—pediatric FOS indication: add-on, all ages (after failure of all other treatments)**					
Articles describing randomized, controlled licensure trials in children could not be found					
**Zonisamide (ZNS)—pediatric FOS indication: add-on**, **≥6 years**					
Brodie et al. (64)	Add-on	≥12 years	351	NA	*≥50% impaired aware FOS responder rate*: PBO: 21.3%; ZNS 500 mg: 52.3%, s
Guerrini et al. (65)	Add-on	6–17 years	207	Unknown: 56.1% Structural: 19.6% Head injuries: 4.7% Family history of epilepsy: 0.9% Other: 18.7%	*≥50% responder rate*: PBO: 31%; ZNS 8 mg/kg: 50%, s

*Add-on, adjunctive therapy; CBZ-CR, controlled-release carbamazepine; FOS, focal-onset seizure; LSM, least square mean; mono, monotherapy; N, number of randomized patients; NA, not available; ns, not statistically significant; PBO, placebo; s, statistically significant*.

*Indications were taken from the summaries of product characteristics available at: https://www.ema.europa.eu/en/medicines, https://mri.cts-mrp.eu/Human/Product/FullTextSearch, or http://www.hpra.ie/homepage/medicines/medicines-information/find-a-medicine/ depending on whether the drug was authorized through a centralized procedure or at national level*.

a *All listed trials had a randomized, placebo-controlled design except where marked otherwise*.

b *Data for the treatment group are provided except if data for the full population were available*.

c *If the study included more than one primary objective, we only included 50% responder rate as this endpoint is currently used for European evaluations*.

d *Secondary outcome (primary outcome was related to cognition)*.

e *Trial identified via information in the product information available at the United States Food and Drug Administration (as information was lacking in European sources)*.

f *Randomized, CBZ-CR-controlled non-inferiority trial*.

g *Randomized, dose-controlled trial*.

h *Subset of children of the indicated age in the intent-to-treat population of the study*.

i *Randomized CBZ- or VPA- and dose-controlled trial*.

### Efficacy Evidence Used for Approval of the Pediatric Indication of ASMs for Focal-Onset Seizures

We aimed to summarize the efficacy data from randomized, controlled trials used to grant approval for the pediatric indication of focal-onset seizures for the ASMs available in Europe. To identify the pivotal studies used for licensure, we consulted the product information made available by the EMA or European national authorities, or—if insufficient information was available through these sources—by the United States Food and Drug Administration. We subsequently used the ClinicalTrials.gov register and PubMed to specifically search for articles that disclosed the results of these studies.

Table 1 summarizes study characteristics and primary efficacy outcomes. Supplementary Table 1 provides additional details on the study design, inclusion/exclusion criteria and other outcomes. Data from pivotal trials in adults are included if the pediatric indication was granted based on extrapolation from adult data.

Consistent with previous literature reviews on the pharmacotherapy of focal-onset seizures and other seizure types in children (80–82), our assessment shows that, among licensure trials, the number of high-quality, randomized, controlled trials performed in children <6 years old is limited, and efficacy data in infants and children <2 or <3 years are nearly absent (Table 1). Many studies included heterogeneous populations, comprising children (or adolescents) as well as adults, and did not analyze efficacy outcomes by age subgroup. Almost no randomized, controlled monotherapy trials have been performed in children with new-onset or untreated focal-onset seizures; instead, most trials assessed adjunctive therapy in patients who did not respond to one or multiple ASMs, and efficacy estimates in these trials were often low (as expected in refractory patients; Table 1, Supplementary Table 1). Moreover, only very few studies provided information on the etiology of epilepsy among enrolled patients, but none showed data segregated by etiology (and would have lacked power to conclude on such subgroup analyses). Likewise, these trials did not investigate specific childhood syndromes.

### Safety Considerations of ASMs in Children and Adolescents

While efficacy data on the pharmacological treatment of focal-onset seizures can be extrapolated from adults to children ≥2 or ≥4 years old, safety and tolerability should be assessed in pediatric clinical trials, with an adequate representation of all age ranges (13, 15, 21, 22). The pediatric safety and tolerability profiles of ASMs have been reviewed extensively (12, 83–87). The most common adverse events associated with ASM use in children and adolescents are similar to those seen in adults and include somnolence, drowsiness, gastrointestinal disturbances, anorexia, irritability, and nervousness (83–85, 88). When choosing the optimal ASM for a patient, certain adverse events may weigh in more at different ages. For instance, weight gain, cosmetic side effects, and depression may be more relevant for older children and adolescents, as are side effects that influence the ability to drive a car (in older adolescents) or teratogenicity (in girls of childbearing potential). Of particular importance to younger children are changes in neurocognitive function (e.g., memory, intelligence, attention, language, and visuomotor coordination) and behavioral effects (e.g., hyperactivity, irritability, agitation, and aggression) (12, 86, 87). However, data from high-quality trials on the effects of ASMs on cognition and behavior are sparse, with conflicting results for some ASMs (12, 86, 87). Heterogeneity across studies in terms of trial design, dose titration, duration of follow-up and methods used to assess cognitive and behavioral outcomes makes it hard to assess and compare the impact of different ASMs (86). A report from the International League Against Epilepsy (ILAE) provided some general recommendations for ASM use in children based on behavioral and cognitive complications (87). Because of the risk of cognitive or behavioral adverse effects, the ILAE recommends careful monitoring of cognition in children receiving phenobarbital, phenytoin, topiramate and zonisamide, and of behavior in children treated with phenobarbital, valproate, gabapentin, topiramate, levetiracetam, and zonisamide. Conversely, the available evidence suggests limited positive effects of lamotrigine on cognition and behavior, and of levetiracetam on cognition (87).

## From Seizure Type-Based to Etiology/Syndrome-Based Research and Treatment

As we concluded based on our overview of pivotal, randomized, controlled trials used for pediatric licensure, most studies evaluate the efficacy of ASMs against seizure types, irrespective of etiology or epilepsy syndrome (Table 1). However, owing to advances in molecular genetics and neuroimaging, there has been a gradual shift from “one-size-fits-all” treatment based on seizure type to more etiology- or syndrome-driven treatment (3, 89–91). An early etiological diagnosis is important, as prompt treatment with adequate drugs (or surgery) may improve the outcome of the child with epilepsy (89, 92–94). In pediatric epilepsy, there are notable examples illustrating the power of etiology- or syndrome-driven research. For example, cannabidiol and fenfluramine were rigorously tested and subsequently approved in Dravet syndrome (95–99), a well-defined epilepsy syndrome with a uniform genetic background. Such research was facilitated as both drugs had received orphan drug designation, which allows sponsors to benefit from incentives established to encourage the development and marketing of drugs to treat rare diseases (100).

In the following sections, we discuss examples of epilepsy syndromes with structural and genetic etiologies characterized by predominantly focal-onset seizures in children. These examples illustrate how etiology- or syndrome-driven research and treatment has been applied or could be considered.

### Structural Etiologies

Structural etiologies may have a genetic basis, as is the case for various cortical malformations, or may be acquired, such as hypoxic-ischemic lesions, brain lesions after accidental or non-accidental trauma, infection, stroke or bleeding (5). In clinical practice, some children with structural epilepsies achieve good long-term seizure control with ASMs. In children with drug-resistant structural epilepsies, surgery is highly effective and should be considered as first-choice treatment in eligible patients (101). However, not all children with structural epilepsies are eligible for surgery, for instance, because of the extent of the epileptogenic zone or the vicinity of the lesion to the eloquent cortex. For structural epilepsies, to date, no dedicated drug trials have been performed. An exception is a study in patients with tuberous sclerosis complex (TSC) (102), a genetic-structural epilepsy with brain tubers causing focal-onset seizures. TSC is caused by mutations in the *TSC1* or *TSC2* gene, which result in overactivation of the mammalian target of rapamycin (mTOR) complex (103). A randomized, controlled trial showed that the mTOR inhibitor everolimus significantly decreased the number of focal-onset seizures in TSC (102, 104). This trial is a good example of etiology-driven research with precision medicine. Everolimus not only affects seizure frequency but also reduces the volume of brain and renal tumors seen in TSC, improves skin lesions and preserves renal function in patients with TSC (105–108).

### Genetic Etiologies

The recognized non-structural focal childhood epilepsy syndromes are mainly genetic or presumed genetic. In most of these epilepsies, treatment is still empiric, purely symptomatic and not always based on results from randomized, controlled trials. However, many of these epilepsies are self-limiting and can be successfully treated, with complete seizure control and the possibility to stop medication after a few years of seizure freedom on medication (109).

In infancy, familial and non-familial epilepsies can be diagnosed (110). The most common genetic etiology for these epilepsies is a mutation in the *PRRT2* gene (proline-rich transmembrane protein 2) (111). Mutations in other genes, e.g., *SCN2A* (sodium voltage-gated channel alpha subunit 2), *KCNQ2* and *KCNQ3* (potassium voltage-gated channel subfamily Q members 2 and 3) have also been identified (110). Seizures are typically focal, with (subtle) behavioral arrest, impaired awareness, automatism and eye/head turning (110), and are usually easily controlled with standard ASMs for focal-onset seizures (112). Although large, prospective trials are lacking, there is increasing evidence that classic sodium channel blockers (SCBs, e.g., carbamazepine) are highly efficacious in potassium channel epilepsies (e.g., due to *KCNQ2* mutations) (112). In *SCN2A* epilepsies, effectiveness of SCBs depends on the mutation type: gain-of-function mutations, typically presenting early in life (<3 months), respond well to SCBs, while loss-of-function mutations, which often have a later onset, are better controlled with other ASMs and may worsen on SCBs (113–115). In SCN8A-related epilepsies, levetiracetam may also be associated with clinical worsening or regression (116). These are typical examples of precision medicine: the precise genetic background of the epilepsy predicts which drugs can be beneficial.

In contrast with these self-limiting syndromes, epilepsy of infancy with migrating focal seizures is usually a drug-resistant epilepsy, within the spectrum of epileptic encephalopathies. Focal seizures start independently in both hemispheres and can “migrate” from one cortical region to another in the same or the other hemisphere, with changing seizure semiology (110). While the cause is often unknown, different gene mutations have been linked with this syndrome. Gain-of-function mutations in *KCNT1* (potassium sodium-activated channel subfamily T member 1) are among the more frequent etiologies (117), and quinidine, a partial KCNT1 antagonist, was tested as precision treatment in this syndrome. Case reports showed seizure reduction in some patients, but this effect was not consistent in all patients (118–122). No randomized, controlled trials have been performed to study the effect of quinidine in *KCNT1*-mutant epilepsy of infancy with migrating focal seizures, and a single randomized, controlled trial in six patients with a different childhood-onset epilepsy syndrome (severe autosomal dominant nocturnal frontal lobe epilepsy) due to *KCNT1* mutations did not demonstrate efficacy (123).

The self-limiting childhood epilepsy syndromes, Panayiotopoulos syndrome and childhood occipital epilepsy (or Gastaut type) are presumed polygenic, but no associated genes have been identified thus far (110). Most patients respond favorably to common ASMs for focal-onset seizures, but no systematic treatment studies have been performed for these epilepsies. By contrast, treatment options for childhood epilepsy with centrotemporal spikes (CECTS), also known as Rolandic epilepsy, one of the best known and most common childhood focal epilepsy syndromes, have been explored in numerous clinical trials (124, 125). CECTS is self-limiting, with an onset between 3 and 14 years of age, characterized by brief, hemifacial seizures that may evolve to focal-to-bilateral tonic-clonic seizures if they occur at night. Seizures resolve during adolescence (110). A few randomized, controlled trials have been performed to assess ASMs in children with CECTS, none of which provided high-quality evidence (81, 124–126). A 2013 evidence review by the ILAE considered carbamazepine and valproate as possibly effective, and gabapentin, levetiracetam, oxcarbazepine and sulthiame as potentially effective monotherapy for CECTS (81). Some low-quality studies have reported that carbamazepine may induce negative myoclonus, atypical absences, drop attacks and electrical status epilepticus during sleep (127). A recent systematic review of seizure freedom rates in patients with CECTS suggested sulthiame, levetiracetam, or clobazam as first-line ASMs for CECTS (125), but highlighted—as in an earlier Cochrane review (124)—that there is insufficient evidence about the optimal ASM for children with CECTS (125). Two randomized, placebo-controlled trials showed that sulthiame (128) or gabapentin (126, 129) monotherapy were effective in preventing seizures in children with CECTS. The other available randomized trials assessing seizure control compared different ASMs [levetiracetam with oxcarbazepine (130, 131), clobazam with carbamazepine (132), topiramate with carbamazepine (133), and levetiracetam with sulthiame (134)]. Most did not show significant differences in seizure control between ASMs, except for one study showing a higher effectiveness of oxcarbazepine vs. levetiracetam (131). A few trials compared the effects of different ASMs on cognition: topiramate vs. carbamazepine (133), clobazam vs. carbamazepine (132), sulthiame vs. levetiracetam (135), and levetiracetam vs. oxcarbazepine (131). Only the levetiracetam-vs.-oxcarbazepine trial found a significant difference between groups, with oxcarbazepine having a superior effect on cognition (131). Low-quality evidence suggests that some children with CECTS do not need treatment with ASM (126). Some experts only consider treatment favorable in case of multiple seizures, early onset, the presence of language dysfunction, cognitive and/or neuropsychological disorder or focal-to-bilateral tonic-clonic seizures (127). Other experts consider that the decision to treat depends on the intensity of interictal epileptiform discharges and their possible negative impact on behavior and cognition (127). Considering the low seizure burden in these self-limiting childhood epilepsy syndromes, high-quality trials are needed to provide evidence on the frequency and type of seizures (i.e., focal aware vs. focal-to-bilateral tonic-clonic) for which ASM treatment should be considered, especially with respect to cognitive outcomes.

## Discussion

With regulators now accepting extrapolation of efficacy data for focal-onset seizures from adults to children ≥2 or ≥4 years of age, the path to licensure of ASMs for this indication in the pediatric population has been simplified, and access to available ASMs can be accelerated. However, the sparsity of high-quality randomized, controlled trials in children, particularly in the very young, and the lack of etiology- or syndrome-specific data in such trials complicates the selection of the optimal ASM. In addition, high-quality data on the effects of ASMs on cognition and behavior are lacking, further complicating treatment decisions. While some childhood epilepsy syndromes respond well to most or several ASMs used for focal-onset seizures, a more etiology- or syndrome-driven approach is needed for others. Improvements in molecular genetics and imaging in recent years have made it possible to determine the etiology of epilepsy in an increasing number of cases, and thereby tailor research and treatment to the patient's needs. For epilepsy syndromes that qualify as rare diseases and drugs that received an orphan designation, various incentives have enabled high-quality randomized, controlled trials. Similar incentives to support research and drug development for more common epilepsy types and syndromes in children might be beneficial and ultimately enable more evidence-based treatment decisions. In addition, long-term follow-up of efficacy and safety in children enrolled in ongoing trials should be stimulated by international collaborations and registries. Study designs such as “N-of-1 trials” (double-blind, randomized crossover trials in single patients, in which the order of experimental and control treatments are randomly allocated to the patient) could provide high-level evidence of approved ASMs and reveal possible new treatment options in rare epilepsies (136). Finally, personalized medicine, or precision medicine, which combines multiple factors (e.g., genetics, age, gender, environment, and lifestyle of individuals) to identify the best way to prevent or treat disease *via* pharmacological and non-pharmacological treatments, may become more common in the future. Different forms of intervention may be embraced, from choosing the most suitable drug according to the type of epilepsy or expected adverse effects to gene therapy. An “ideal” epilepsy therapy would stop seizures and undo the changes caused by specific genetic mutations (137, 138).

## Author Contributions

AA and LL: conceptualization, investigation, writing—original draft, and writing—review and editing. BC: conceptualization, investigation, and writing—review and editing. All authors contributed to the article and approved the submitted version.

## Funding

This work received funding from Eisai Co., Ltd.

## Conflict of Interest

AA declares having received fees from Eisai and Zogenix for participating in advisory boards and support from UCB and Zogenix for attending meetings. BC declares that Antwerp University Hospital may benefit financially from a royalty arrangement that is related to this research if Zogenix is successful in marketing its product, fenfluramine and also declares having received fees from Eisai, Brabant Pharma, Novartis, UCB, and Zogenix for participating in advisory boards. LL declares having received consultancy fees and payment or honoraria for lectures, presentations, speakers bureaus, manuscript writing, or educational events from Novartis, Zogenix, Eisai, LivaNova, UCB, and Epihunter, and in addition having a patent for the use of fenfluramine with potential royalties paid through the University of Leuven. The authors declare that this work received funding from Eisai Co., Ltd. The funder had the following involvement with the work: payment for medical writing services and publishing costs.

## Publisher's Note

All claims expressed in this article are solely those of the authors and do not necessarily represent those of their affiliated organizations, or those of the publisher, the editors and the reviewers. Any product that may be evaluated in this article, or claim that may be made by its manufacturer, is not guaranteed or endorsed by the publisher.

## Abbreviations

ASM: antiseizure medication
CECTS: childhood epilepsy with centrotemporal spikes
EMA: European Medicines Agency
ILAE: International League Against Epilepsy
KCNQ2/KCNQ3: potassium voltage-gated channel subfamily Q members 2 and 3
KCNT1: potassium sodium-activated channel subfamily T member 1
mTOR: mammalian target of rapamycin
PRRT2: proline-rich transmembrane protein 2
SCB: sodium channel blocker
SCN2A: sodium voltage-gated channel alpha subunit 2
TSC: tuberous sclerosis complex.
