# Supplementary material for: Treatment of Focal-Onset Seizures in Children: Should This Be More Etiology-Driven?
Source: Front Neurol. 2022 Mar 7;13:842276. doi: 10.3389/fneur.2022.842276 (PMC8940242; doi:10.3389/fneur.2022.842276)
Supplement: Supplementary file 1 [file Table_1.DOCX]

**Supplementary table 1. Summary of efficacy evidence used as basis for the pediatric indication of antiseizure medication for the treatment of focal-onset seizures**

| **Reference** | **Trial design** | **Inclusion/exclusion criteria^a^** | **Efficacy outcome^a^** |
| --- | --- | --- | --- |
| **Brivaracetam** | | | |
| (1) | Randomized, PBO-controlled, double-blind, add-on  8-week baseline,  12-week treatment (BRV 5, 20, 50 mg/day),  1-week taper or entry in LTFU | INCL: 16–70 years, uncontrolled FOS, 1–2 ASMs (≤20% LEV), ≥2 FOS/month in 3-month pre-screening, ≥8 FOS in 8-week baseline  EXCL: non-motor aware FOS only, cluster seizures, status epilepticus, pseudo-seizures, rapidly progressing brain disorder/tumor | *% reduction vs PBO in weekly FOS frequency:*  BRV 5 mg: -0.9%, ns  BRV 20 mg: 4.1%, ns  BRV 50 mg: 12.8%, s  *Median % reduction vs baseline in weekly FOS frequency:*  PBO: 17.8%  BRV 5 mg: 20.0%, ns  BRV 20 mg: 22.5%, ns  BRV 50 mg: 30.5%, s  *≥50% FOS responder rate:*  PBO: 16.7%  BRV 5 mg: 21.9%, ns  BRV 20 mg: 23.2%, ns  BRV 50 mg: 32.7%, s  *Seizure freedom (all types)*:  PBO: 0.0%  BRV 5 mg: 1.1%  BRV 20 mg: 1.0%  BRV 50 mg: 4.0% |
| (2) | Randomized, PBO-controlled, double-blind, add-on  8-week baseline,  12-week treatment (20, 50, 100 mg/day),  2-week taper or entry in LTFU | INCL: 16–70 years, uncontrolled FOS or epileptic syndrome, 1–2 ASMs (≤20% LEV), ≥2 FOS/month in 3-month pre-screening, ≥8 FOS in 8-week baseline  EXCL: non-motor aware FOS only, cluster seizures, status epilepticus, pseudo-seizures, rapidly progressing brain disorder/tumor | *% reduction vs PBO in weekly FOS frequency:*  BRV 20 mg: 6.8%, ns  BRV 50 mg: 6.5%, ns  BRV 100 mg: 11.7%, s  *Median % reduction vs baseline in weekly FOS frequency:*  PBO: 17.0%  BRV 20 mg: 30.0%, s  BRV 50 mg: 26.8%, ns  BRV 100 mg: 32.5%, s  *≥50% FOS responder rate:*  PBO: 20.0%  BRV 20 mg: 27.3%, ns  BRV 50 mg: 27.3%, ns  BRV 100 mg: 36.0%, s  *Seizure freedom (all types)*:  PBO: 0.0%  BRV 20 mg: 2.0%  BRV 50 mg: 0.0%  BRV 100 mg: 4.0% |
| (3) | Randomized, PBO-controlled, double-blind, add-on  8-week baseline,  12-week treatment (100, 200 mg/day),  4-week taper or entry in LTFU | INCL: 16–80 years, uncontrolled FOS or epileptic syndrome, 1–2 ASMs (no LEV), ≥2 FOS/month in 3-month pre-screening, ≥8 FOS in 8-week baseline  EXCL: non-motor aware FOS only, cluster seizures, status epilepticus, psychogenic non-epileptic seizures, rapidly progressing brain disorder/tumor | *≥50% FOS responder rate:*  PBO: 21.6%  BRV 100 mg: 38.9%, s  BRV 200 mg: 37.8%, s  *% reduction vs PBO in 4-week FOS frequency:*  BRV 100 mg: 22.8%, s  BRV 200 mg: 23.2%, s  *Median % reduction vs baseline in FOS frequency:*  PBO: 17.6%  BRV 100 mg: 37.2%, s  BRV 200 mg: 35.6%, s  *Seizure freedom (all types)*:  PBO: 0.8%  BRV 100 mg: 5.2%, s  BRV 200 mg: 4.0%, s |
| **Eslicarbazepine acetate** | | | |
| (4) | Randomized, PBO-controlled, double-blind, add-on  8-week baseline,  2-week titration,  12-week maintenance (ESL 400, 800, 1200 mg/day),  4-week taper | INCL: ≥18 years, uncontrolled FOS, 1–2 ASMs (no FBM or OXC), ≥4 FOS per 4-week baseline  EXCL: non-motor aware FOS only, GOS, rapid progressive neurologic disorder, cluster seizures, status epilepticus, psychogenic seizures | *LSM log difference vs PBO in 4-week seizure frequency:*  ESL 400 mg: -0.08, ns  ESL 800 mg: -0.19, s  ESL 1200 mg: -0.22, s  *Median % reduction vs baseline in seizure frequency:*  PBO: 16%  ESL 400 mg: 26%  ESL 800 mg: 36%  ESL 1200 mg: 45%  *≥50% responder rate:*  PBO: 20%  ESL 400 mg: 23%, ns  ESL 800 mg: 34%, s  ESL 1200 mg: 43%, s  *Seizure freedom*:  PBO: 2%  ESL 400 mg: 2%, ns  ESL 800 mg: 4%, ns  ESL 1200 mg: 8%, s |
| (5) | Randomized, PBO-controlled, double-blind, add-on  8-week baseline,  2-week titration,  12-week maintenance (ESL 800, 1200 mg/day),  4-week taper,  entry in LTFU | INCL: ≥18 years, uncontrolled FOS, 1–2 ASMs (no FBM or OXC), ≥4 FOS per 4-week pre-screening and per 4-week baseline  EXCL: non-motor aware FOS only, GOS, rapid progressive neurologic disorder, cluster seizures, status epilepticus, psychogenic seizures | *LSM difference vs PBO in 4-week seizure frequency:*  ESL 800 mg: -1.6, s  ESL 1200 mg: -1.9, s  *≥50% responder rate:*  PBO: 22.6%  ESL 800 mg: 34.5%, ns  ESL 1200 mg: 37.7%, s  *Median % reduction vs baseline in seizure frequency:*  PBO: 17.0%  ESL 800 mg: 37.9%  ESL 1200 mg: 41.9%  *Seizure freedom*:  PBO: 1.2%  ESL 800 mg: 4.8%, ns  ESL 1200 mg: 3.9%, ns |
| (6) | Randomized, PBO-controlled, double-blind, add-on  8-week baseline,  14-week treatment (ESL 400, 800, 1200 mg/day – 2-week titration for 1200 mg dose),  entry in LTFU | INCL: ≥18 years, uncontrolled FOS, 1–3 ASMs (no FBM or OXC), ≥4 FOS per 4-week pre-screening and per 4-week baseline  EXCL: non-motor aware FOS only, GOS, rapid progressive neurologic disorder, cluster seizures, status epilepticus, psychogenic seizures | *LSM difference vs PBO in 4-week seizure frequency:*  ESL 400 mg: -1.5, ns  ESL 800 mg: -3.8, s  ESL 1200 mg: -3.5, s  *≥50% responder rate:*  PBO: 13.0%  ESL 400 mg: 16.7%, ns  ESL 800 mg: 40.0%, s  ESL 1200 mg: 37.1%, s  *Median % reduction vs baseline in seizure frequency:*  PBO: 0.8%  ESL 400 mg: 18.7%, ns  ESL 800 mg: 32.6%, s  ESL 1200 mg: 32.8%, s  *Seizure freedom*:  PBO: 1.0%  ESL 400 mg: 1.0%, ns  ESL 800 mg: 8.0%, s  ESL 1200 mg: 4.1%, ns |
| (7) | Randomized, PBO-controlled, double-blind, add-on  8-week baseline,  2-week titration,  12-week maintenance (ESL 800, 1200 mg/day),  2-week taper or entry in LTFU | INCL: ≥16 years, uncontrolled FOS, 1–2 ASMs (no OXC), ≥4 FOS in 4-week pre-screening, ≥8 FOS in 8-week baseline  EXCL: non-motor aware FOS only, GOS, progressive neurologic disorder, cluster seizures, status epilepticus, psychogenic or non-epileptic seizures | *LSM log difference vs PBO in 4-week seizure frequency:*  ESL 800 mg: -0.18, ns  ESL 1200 mg: -0.26, s  *≥50% responder rate:*  PBO: 23.1%  ESL 800 mg: 30.5%, ns  ESL 1200 mg: 42.6%, s  *Median % reduction vs baseline in seizure frequency:*  PBO: 21.8%  ESL 800 mg: 29.7%, ns  ESL 1200 mg: 35.6%, s  *Seizure freedom*:  PBO: 0.9%  ESL 800 mg: 2.0%  ESL 1200 mg: 2.2% |
| (8) | Randomized, PBO-controlled, double-blind, add-on  8-week baseline,  6-week titration,  12-week maintenance (ESL 20 mg/kg/day),  4-week taper, 4-week follow-up,  entry in LTFU | INCL: 2–18 years, uncontrolled FOS, 1–2 ASMs (no OXC), ≥4 FOS in 1-month pre-screening  EXCL: GOS, progressive neurological disorder, status epilepticus, non-epileptic seizures, Lennox-Gastaut, West syndrome | ≥*50% responder rate*:  PBO: 31.0%  ESL 20 mg/kg: 30.6%, ns  *Mean % reduction in seizure frequency vs baseline*:  PBO: 9.6%  ESL 20 mg/kg: 19.2%, ns |
| (9) | Randomized, PBO-controlled, double-blind, add-on  4-week baseline,  4-week titration,  8-week maintenance (ESL 30 mg/kg/day),  4-week taper, 4-week follow-up or entry in LTFU | INCL: 6–16 years, uncontrolled FOS, 1–2 ASMs (no OXC), ≥4 FOS in 1-month pre-screening  EXCL: non-motor aware FOS only, GOS, progressive neurologic disorder, cluster seizures, status epilepticus, psychogenic seizures, Lennox-Gastaut, West syndrome | *≥50% responder rate:*  PBO: 25.0%  ESL 30 mg/kg: 50.6%, s  *Median % reduction in seizure frequency vs baseline:*  PBO: 31.6%  ESL 30 mg/kg: 51.7%, s  *Seizure freedom:*  PBO: 5.0%  ESL 30 mg/kg: 25.3%, s |
| **Gabapentin** | | | |
| (10) | Randomized, PBO-controlled, double-blind, add-on  3-month baseline,  2-week titration,  12-week maintenance (GBP 1200 mg/day) | INCL: 14–73 years (actual range), uncontrolled FOS, 1–2 ASMs, ≥1 FOS per week baseline | *≥50% FOS responder rate:*  PBO: 9%  GBP 1200 mg: 23%, s  *Median % reduction in FOS frequency vs baseline:*  PBO: 12.5%  GBP 1200 mg: 29.2% |
| (11) | Randomized, PBO-controlled, double-blind, add-on  12-week baseline,  12-week treatment,  (GBP 600, 1200, 1800 mg/day – incl. 2–3-day titration) | INCL: ≥16 years, uncontrolled FOS, 1–2 ASMs, ≥4 FOS/month in 3-month pre-screening, ≥6 FOS in 12-week baseline  EXCL: atypical absence seizures, non-epileptic seizures, progressive structural CNS lesion or encephalopathy | *≥50% FOS responder rate:*  PBO: 8.4%  GBP 600 mg: 18.4%, ns  GBP 1200 mg: 17.6%, ns  GBP 1800 mg: 26.4%, s  *Median % reduction in FOS frequency vs baseline:*  PBO: 5.9%  GBP 600 mg: 24.3%  GBP 1200 mg: 20.0%  GBP 1800 mg: 31.9% |
| (12) | Randomized, PBO-controlled, double-blind, add-on  12-week baseline,  12-week treatment (GBP 900, 1200 mg/day – incl. 2-day titration),  optional 4-week follow-up and entry in LTFU | INCL: ≥12 years, uncontrolled FOS, 1–2 ASMs, ≥4 FOS/month in 3-month pre-screening, ≥6 FOS in 12-week baseline  EXCL: atypical absence seizures, non-epileptic seizures, progressive structural CNS lesions | *Median % reduction in FOS frequency vs baseline:*  PBO: 0.3%  GBP 900 mg: 21.8%  GBP 1200 mg: 17.8%  *≥50% FOS responder rate:*  PBO: 10.1%  GBP 900 mg: 22.9%, s  GBP 1200 mg: 28.0%, s |
| (13) | Randomized, PBO-controlled, double-blind, add-on  6-week baseline,  12-week treatment (GBP 23–35 mg/kg/day – incl. 2–3-day titration),  4-week FU or entry in LTFU | INCL: 3–12 years, uncontrolled FOS, 1–3 ASMs, ≥4 FOS in 6-week baseline  EXCL: absence seizures, seizures related to drugs, alcohol or acute medical illness, progressive structural CNS lesions or encephalopathies, benign epilepsy syndromes | *LSM of response ratio for FOS:*  PBO: -0.072  GBP 23–35 mg/kg: -0.161, s  *≥50% FOS responder rate:*  PBO: 17.5%  GBP 23–35 mg/kg: 21.2%, ns  *Median % reduction in FOS frequency vs baseline:*  PBO: 6.5%  GBP 23–35 mg/kg: 17.0%  *Seizure freedom:*  PBO: 0.8%  GBP 23–35 mg/kg: 2.7% |
| **Lacosamide** | | | |
| (14) | Randomized, PBO-controlled, double-blind, add-on  8-week baseline,  6-week titration,  12-week maintenance (LCM 200, 400, 600 mg/day),  3-week taper or entry in LTFU | INCL: 18–65 years, uncontrolled FOS, 1–2 ASMs (no FBM or VGB), ≥4 FOS per 4-week baseline  EXCL: GOS, cluster seizures, status epilepticus, non-epileptic or psychogenic seizures, progressive structural CNS lesion or encephalopathy | *≥50% responder rate:*  PBO: 21.9%  LCM 200 mg: 32.7%, ns  LCM 400 mg: 41.1%, s  LCM 600 mg: 38.1%, s  *Median % reduction in seizure frequency vs baseline:*  PBO: 10%  LCM 200 mg: 26%, ns  LCM 400 mg: 39%, s  LCM 600 mg: 40%, s  *Seizure freedom:*  PBO: 0.0%  LCM 200 mg: 0.9%  LCM 400 mg: 4.7%  LCM 600 mg: 1.0% |
| (15) | Randomized, PBO-controlled, double-blind, add-on  8-week baseline,  4-week titration,  12-week maintenance (LCM 200, 400 mg/day),  2-week taper or entry in LTFU | INCL: 16–70 years, uncontrolled FOS, 1–3 ASMs (no FBM or VGB), ≥4 FOS per 4-week pre-screening and baseline  EXCL: GOS, cluster seizures, status epilepticus, non-epileptic or psychogenic seizures, progressive structural CNS lesion or encephalopathy | *≥50% FOS responder rate:*  PBO: 25.8%  LCM 200 mg: 35.0%, ns  LCM 400 mg: 40.5%, s  *Median % reduction in FOS frequency vs baseline:*  PBO: 20.5%  LCM 200 mg: 35.3%, s  LCM 400 mg: 36.4%, s  *Seizure freedom:*  PBO: 2.1%  LCM 200 mg: 3.6%  LCM 400 mg: 2.4% |
| (16) | Randomized, PBO-controlled, double-blind, add-on  8-week baseline,  6-week titration,  12-week maintenance (LCM 400, 600 mg/day),  3-week taper or entry in LTFU | INCL: 16–70 years, uncontrolled FOS, 1–3 ASMs (no FBM or VGB), ≥4 FOS per 4-week pre-screening and baseline  EXCL: GOS, cluster seizures, status epilepticus, non-epileptic or psychogenic seizures | *≥50% FOS responder rate:*  PBO: 18.3%  LCM 400 mg: 38.3%, s  LCM 600 mg: 41.2%, s  *Median % reduction in FOS frequency vs baseline:*  PBO: 20.8%  LCM 400 mg: 37.3%, s  LCM 600 mg: 37.8%, s  *Seizure freedom:*  PBO: 0.0%  LCM 400 mg: 2.5%  LCM 600 mg: 8.1% |
| (17) | Randomized, PBO-controlled, double-blind, add-on  8-week baseline,  6-week titration,  10-week maintenance (6–12 mg/kg/day),  4-week taper/30-day follow-up or entry in LTFU | INCL: 4–16 years, uncontrolled FOS; 1–3 ASMs, ≥2 FOS per 4-week pre-screening and 8-week baseline  EXCL: GOS, mixed seizure disorder (FOS + GOS), status epilepticus; Lennox-Gastaut, exclusively febrile or nocturnal seizures, epilepsy secondary to progressive cerebral or neurodegenerative disease | *Median % reduction in FOS frequency vs baseline:*  PBO: 21.7%  LCM 6–12 mg/kg: 51.7%  *% reduction vs PBO in FOS frequency:*  LCM 6–12 mg/kg: 31.7%, s  *≥50% FOS responder rate:*  PBO: 33.3%  LCM 6–12 mg/kg: 52.9%, s  *Seizure freedom:*  PBO: 9.7%  LCM 6–12 mg/kg: 15.1% |
| (18) | Randomized, CBZ-CR-controlled, double-blind, non-inferiority, mono  2-week titration,  1-week stabilization,  6-month assessment,  6-month maintenance  start on lowest dose (LCM 200 mg/day; CBZ-CR 400 mg/day),  if seizure during assessment, then next dose (LCM 400 mg/day; CBZ-CR 800 mg/day): new 6-month assessment, 6-month maintenance;  if seizure during assessment for this dose, then highest dose (LCM 600 mg/day; CBZ-CR 1200 mg/day): new 6-month assessment, 6-month maintenance,  ≥7-week end of trial phase or entry in LTFU | INCL: ≥16 years, ≥2 FOS or GTCS in year pre-screening (≥1 in 3-month pre-screening)  EXCL: idiopathic generalized epilepsy, seizures other than FOS or GTCS, cluster seizures, status epilepticus, non-epileptic seizures, conversion disorders | *Kaplan-Meier % of patients 6 months seizure free (last assessed dose):*  CBZ-CR 400–1200 mg: 91.1%  LCM 200–600 mg: 89.8%, non-inferiority met  *Kaplan-Meier % of patients 12 months seizure free (last assessed dose):*  CBZ-CR 400–1200 mg: 83%  LCM 200–600 mg: 78% |
| **Lamotrigine** | | | |
| (19) | Randomized, PBO-controlled, double-blind, add-on  8-week baseline,  6-week titration,  12-week maintenance (LTG 1–15 mg/kg/day depending on concurrent ASM),  1–6-week taper or entry in LTFU | INCL: 2–16 years, uncontrolled FOS, 1–2 ASMs (no FBM or GBP), ≥4 FOS expected per 4-week baseline  EXCL: GOS, pseudo-seizures, drug-induced or metabolic seizures, status epilepticus, intracerebral structural lesions, progressive neurological disorder | *Median % reduction in FOS frequency vs baseline:*  PBO: 6.7%  LTG 1–15 mg/kg: 36.1%, s  *≥50% FOS responder rate:*  PBO: 16%  LTG 1–15 mg/kg: 42%, s |
| (20) | Randomized, PBO-controlled, double-blind, responder-enriched, add-on  open-label phase LTG add-on (LTG max 5.1–15.6 mg/kg/day depending on concurrent ASM),  responders enter 8-week randomized double-blind phase (LTG vs withdrawal from LTG to PBO) | INCL: 1–24 months, uncontrolled FOS, 1 ASM (no FBM), ≥4 FOS per month, ≥40% reduction in FOS frequency in open-label phase  EXCL: progressive myoclonic epilepsy, progressive neurologic disease, seizures unrelated to epilepsy or resulting from drug withdrawal | *% treatment failure*:  PBO: 84%  LTG 5.1–15.6 mg/kg: 58%, ns |
| **Levetiracetam** | | | |
| (21) | Randomized, PBO-controlled, double-blind, add-on  8-week baseline,  4-week titration,  10-week maintenance (LEV 60 mg/kg/day),  6-week taper or entry in LTFU | INCL: 4–16 years, uncontrolled FOS; 1–2 ASMs (no FBM), ≥4 FOS in 4-week pre-screening and per 4-week baseline  EXCL: treatable seizure etiology, epilepsy secondary to progressive cerebral or neurodegenerative disease, pseudo-seizures, Lennox-Gastaut, cluster seizures, status epilepticus | *% reduction vs PBO in weekly FOS frequency:*  LEV 60 mg/kg: 26.8%, s  *Median % reduction in FOS frequency vs baselin*e:  PBO: 16.3%  LEV 60 mg/kg: 43.3%, s  *≥50% FOS responder rate*:  PBO: 19.6%  LEV 60 mg/kg: 44.6%, s  *Seizure freedom*:  PBO: 1.0%  LEV 60 mg/kg: 6.9% |
| (22) | Randomized, PBO-controlled, double-blind, add-on  2-day baseline,  1-day titration,  4-day maintenance (LEV 40–50 mg/kg/day),  2-week taper or entry in LTFU | INCL: 1 month–3 years, uncontrolled FOS, 1–2 ASMs, ≥2 FOS/week in 2-week pre-screening, ≥2 FOS in 2-day baseline  EXCL: treatable seizure etiology (metabolic, toxic, infectious disorders, febrile seizures), pseudo-seizures, Lennox-Gastaut, status epilepticus, epilepsy secondary to progressive cerebral or neurodegenerative disease | *≥50% FOS responder rate*:  PBO: 19.6%  LEV 40–50 mg/kg: 43.1%, s  *Median % reduction in daily FOS frequency vs baselin*e:  PBO: 7.1%  LEV 40–50 mg/kg: 43.6%, s  *Seizure freedom:*  PBO: 5.9%  LEV 40–50 mg/kg: 15.5% |
| (23) | Randomized, CBZ-CR-controlled, double-blind non-inferiority, mono  2-week titration,  1-week stabilization,  6-month assessment,  6-month maintenance,  start with lowest dose (LEV 1000 mg/day; CBZ-CR 400 mg/day),  if seizure during assessment, then next dose (LEV 2000 mg/day; CBZ-CR 800 mg/day): new 6-month assessment, 6-month maintenance,  if seizure during assessment for this dose, then highest dose (LEV 3000 mg/day; CBZ-CR 1200 mg/day): new 6-month assessment, 6-month maintenance | INCL: ≥16 years, ≥2 FOS or GTCS in year pre-screening (≥1 in 3-month pre-screening)  EXCL: seizures other than FOS or GTCS, cluster seizures, idiopathic generalized epilepsy | *% patients 6 months seizure free (last assessed dose):*  CBZ-CR: 72.8%  LEV: 73.0%, non-inferiority met  *% patients 12 months seizure free (last assessed dose):*  CBZ-CR: 58.5%  LEV: 56.6% |
| **Oxcarbazepine** | | | |
| (24) | Randomized, PBO-controlled, double-blind, add-on  8-week baseline,  2-week titration,  24-week maintenance (OXC 600, 1200, 2400 mg/day),  2-week taper,  entry in LTFU | INCL: 15–65 years, uncontrolled FOS, 1–3 ASMs (no ETS or FBM), ≥4 FOS/month in 8-week baseline  EXCL: seizures of metabolic, neoplastic or infectious origin, status epilepticus, progressive neurological disorder | *Median % reduction in seizure frequency vs baselin*e:  PBO: 7.6%  OXC 600 mg: 26.5%, s  OXC 1200 mg: 40.2%, s  OXC 2400 mg: 50.0%, s  *≥50% responder rate*:  PBO: 12.7%  OXC 600 mg: 26.8%, s  OXC 1200 mg: 41.2%, s  OXC 2400 mg: 50.0%, s  *Seizure freedom:*  PBO: 0.6%  OXC 600 mg: 3%  OXC 1200 mg: 10%  OXC 2400 mg: 22% |
| (25) | Randomized, PBO-controlled, double-blind, add-on  56-day baseline,  14-day titration,  98-day maintenance (OXC 30–46 mg/kg/day),  entry in LTFU | INCL: 3–17 years, uncontrolled FOS, 1–2 ASMs (no FBM), ≥8 FOS in 56-day baseline  EXCL: progressive lesion, status epilepticus, seizures of metabolic, neoplastic or active infectious origin, progressive neurologic disorder | *Median % reduction in FOS frequency vs baselin*e:  PBO: 9%  OXC 30–46 mg/kg: 35%, s  *≥50% FOS responder rate*:  PBO: 22%  OXC 30–46 mg/kg: 41%, s  *Seizure freedom:*  PBO: 0.8%  OXC 30–46 mg/kg: 3.7% |
| (26) | Randomized, dose-controlled, observer-blind, add-on  24–72-hour baseline,  9-day maintenance (10 mg/kg/day) or  26-day titration and 9-day maintenance (60 mg/kg/day),  entry in LTFU | INCL: 1 month–3 years, uncontrolled FOS (severe epileptic syndromes), 1–2 ASMs, ≥2 FOS during baseline  EXCL: treatable etiology of seizures, GOS, cluster seizures, status epilepticus, evolving space-occupying lesion or progressive neurologic disease | *Median absolute reduction in type 1 seizure frequency:*  OXC 10 mg/kg: 1.4  OXC 60 mg/kg: 2.0, s  *Median % reduction in type 1 seizure frequency:*  OXC 10 mg/kg: 46.2%  OXC 60 mg/kg: 83.3%, s  *≥50% responder rate*:  OXC 10 mg/kg: 47%  OXC 60 mg/kg: 64% |
| (27) | Randomized, PBO-controlled, double-blind, mono  2-day screening,  1-day titration,  9-day maintenance (OXC 2400 mg/day),  entry in LTFU | INCL: 11–65 years, 2–10 FOS in 2-day screening, no ASMs in 2-day screening (except lorazepam up to 8 mg/day)  EXCL: status epilepticus, non-epileptic seizures, progressive cerebral lesion, seizures of metabolic, neoplastic or active infectious origin, progressive neurological disorder | *Time to exit:*  Significantly in favor of OXC 2400 mg vs PBO  *Exit rate:*  PBO: 84%  OXC: 47%, s  *Seizure freedom:*  PBO: 2%  OXC: 25% |
| (28) | Randomized, dose-controlled, double-blind, CBZ-to-OXC conversion, mono  56-day screening (CBZ 800–1600 mg/day),  28-day conversion,  56-day baseline (OXC 2400 mg/day),  126-day treatment (OXC 300 mg, 2400 mg/day),  entry in LTFU | INCL: ≥12 years, FOS uncontrolled on CBZ, 2–40 FOS per 28-day screening  EXCL: space-occupying lesions, status epilepticus, seizures of metabolic, toxic or active infectious origin, non-epileptic seizures | *Time to exit:*  Significantly in favor of OXC 2400 mg vs 300 mg  OXC 300 mg: 28 days  OXC 2400 mg: 68 days  *Exit rate:*  OXC 300 mg: 100%  OXC 2400 mg: 65%, s |
| (29) | Randomized, dose-controlled, double-blind, ASM-to-OXC conversion, mono  56-day baseline,  126-day treatment (OXC 300, 2400 mg/day),  entry in LTFU | INCL: ≥12 years, uncontrolled FOS, 1–2 ASMs (no FBM), 2–40 FOS per 28-day baseline  EXCL: aware FOS only, progressive lesion, status epilepticus, non-epileptic seizures | *Exit rate:*  OXC 300 mg: 93.3%  OXC 2400 mg: 41.2%, s  *Time to exit:*  Significantly in favor of OXC 2400 mg vs 300 mg  *≥50% responder rate:*  OXC 300 mg: 7%  OXC 2400 mg: 42%  *Seizure freedom:*  OXC 300 mg: 0%  OXC 2400 mg: 12% |
| **Perampanel** | | | |
| (30) | Randomized, PBO-controlled, double-blind, add-on  6-week baseline,  6-week titration,  13-week maintenance (PER 8, 12 mg/day),  4-week follow-up or entry in LTFU | INCL: ≥12 years, uncontrolled FOS, 1–3 ASMs (no VGB), ≥5 FOS in 6-week baseline  EXCL: non-motor aware FOS only, GOS, Lennox-Gastaut, cluster seizures, status epilepticus, psychogenic seizures, progressive CNS disease | *≥50% responder rate*:  PBO: 26.4%  PER 8 mg: 37.6%, ns  PER 12 mg: 36.1%, ns  *Median % reduction in seizure frequency vs baselin*e:  PBO: 21.0%  PER 8 mg: 26.3%, s  PER 12 mg: 34.5%, s  *Seizure freedom*:  PBO: 0.0%  PER 8 mg: 2.2%  PER 12 mg: 1.5% |
| (31) | Randomized, PBO-controlled, double-blind, add-on  6-week baseline,  6-week titration,  13-week maintenance (PER 8, 12 mg/day),  4-week follow-up or entry in LTFU | INCL: ≥12 years, uncontrolled FOS, 1–3 ASMs (no VGB), ≥5 FOS in 6-week baseline  EXCL: non-motor aware FOS only, GOS, Lennox-Gastaut, cluster seizures, status epilepticus, non-epileptic or psychogenic seizures, progressive CNS disease | *≥50% responder rate*:  PBO: 14.7%  PER 8 mg: 33.3%, s  PER 12 mg: 33.9%, s  *Median % reduction in seizure frequency vs baselin*e:  PBO: 9.7%  PER 8 mg: 30.5%, s  PER 12 mg: 17.6%, s  *Seizure freedom*:  PBO: 1.5%  PER 8 mg: 2.3%  PER 12 mg: 5.0% |
| (32) | Randomized, PBO-controlled, double-blind, add-on  6-week baseline,  6-week titration,  13-week maintenance (PER 2, 4, 8 mg/day),  4-week follow-up or entry in LTFU | INCL: ≥12 years, uncontrolled FOS, 1–3 ASMs (no VGB), ≥5 FOS in 6-week baseline  EXCL: non-motor aware FOS only, GOS, Lennox-Gastaut, cluster seizures, status epilepticus, psychogenic seizures, progressive CNS disease | *≥50% responder rate*:  PBO: 17.9%  PER 2 mg: 20.6%, ns  PER 4 mg: 28.5%, s  PER 8 mg: 34.9%, s  *Median % reduction in seizure frequency vs baselin*e:  PBO: 10.7%  PER 2 mg: 13.6%, ns  PER 4 mg: 23.3%, s  PER 8 mg: 30.8%, s  *Seizure freedom*:  PBO: 1.2%  PER 2 mg: 1.9%  PER 4 mg: 4.4%  PER 8 mg: 4.8% |
| **Tiagabine** | | | |
| (33) | Randomized, PBO-controlled, double-blind, add-on  12-week baseline,  4-week titration,  12-week maintenance (TGB 16, 32, 56 mg/day)  4-week taper | INCL: 12–77 years, uncontrolled impaired aware FOS, 1–3 hepatic enzyme-inducers (VPA allowed in combination with hepatic enzyme-inducer), ≥6 impaired aware FOS in 8-week pre-screening, ≥8 impaired aware FOS in 12-week baseline  EXCL: progressive lesions | *Median % reduction in impaired aware FOS frequency vs baseline*:  PBO: 11%  TGB 16 mg: 13%, ns  TGB 32 mg: 25%, s  TGB 56 mg: 33%, s  *≥50% impaired aware FOS responder rate*:  PBO: 4%  TGB 16 mg: 8%, ns  TGB 32 mg: 20%, s  TGB 56 mg: 29%, s |
| (34) | Randomized, PBO-controlled, double-blind, add-on  8-week baseline,  4-week titration,  8-week maintenance (TGB 2x16, 4x8 mg/day),  4-week termination or entry in LTFU | INCL: 12–75 years, uncontrolled impaired aware FOS, 1–3 ASMs (≥1 hepatic enzyme inducer), ≥6 impaired aware FOS in 8-week pre-screening  EXCL: pseudo-seizures, active CNS disease | *Median absolute reduction in impaired aware FOS frequency vs baseline*:  TGB 2x16 mg: 1.6, ns  TGB 4x8 mg: 1.2, s  *≥50% impaired aware FOS responder rate*:  PBO: 10%  TGB 2x16 mg: 31%, s  TGB 4x8 mg: 27%, s |
| **Topiramate** | | | |
| (35) | Randomized, PBO-controlled, double-blind, add-on  8-week baseline,  8-week titration,  8-week maintenance (TPM 6 mg/kg/day) | INCL: 2–16 years, uncontrolled FOS, 1–2 ASMs, ≥6 FOS in 8-week baseline  EXCL: treatable cause of seizures, progressive CNS disorder, Lennox-Gastaut, status epilepticus | *% reduction in FOS frequency vs baseline*:  PBO: 10.5%  TPM 6 mg/kg: 33.1%, s  *≥50% FOS responder rate*:  PBO: 20%  TPM 6 mg/kg: 39%, ns |
| (36)  (37) | Randomized, dose-controlled, double-blind, mono  3-month retrospective baseline,  7-day open-label (TPM 25 mg/day),  ≥6-month treatment  (TPM 50, 400 mg/day) | INCL: 6–15 years, 1–2 FOS or GTCS in 3-month retrospective baseline  EXCL: (atypical) absence seizures, epilepsia partialis continua, myoclonic seizures only, cluster or serial seizures, non-epileptic seizures, neurodegenerative disease | *Time to first seizure*  Significantly longer with TPM 400 mg than TPM 50 mg  *Kaplan-Meier % patients 6 months seizure free*:  TPM 50 mg: 78%  TPM 400 mg: 90%, s  *Kaplan-Meier % patients 12 months seizure free*:  TPM 50 mg: 62%  TPM 400 mg: 85%, s |
| (38)  (39) | Randomized, CBZ/VPA- and dose-controlled, double-blind, mono  3-month retrospective baseline,  ≥6-month treatment  (TPM 100, 200 mg/day, CBZ 600 mg/day, VPA 1250 mg/day) | INCL: 6–16 years, FOS, GOS or unclassified  EXCL: non-epileptic seizures, treatable cause of seizures, progressive cerebral lesion | *Time to exit*:  No significant difference between groups  *Time to first seizure (any type)*:  No significant difference between groups  *Seizure freedom (last 6 months, any type)*:  CBZ: 39%  VPA: 53%  TPM 100 mg: 63%  TPM 200 mg: 59% |
| **Zonisamide** | | | |
| (40) | Randomized, PBO-controlled, double-blind, add-on  12-week baseline,  6-week titration,  18-week maintenance (ZNS 100, 300, 500 mg/day),  4 -week taper or entry in LTFU | INCL: ≥12 years, uncontrolled FOS, 1–3 ASMs, ≥12 FOS in 12-week baseline  EXCL: non-epileptic seizures, progressive neurologic disease | *≥50% impaired aware FOS responder rate*:  PBO: 21.3%  ZNS 500 mg: 52.3%, s  *% reduction in impaired aware FOS frequency vs baseline*:  PBO: 16.3%  ZNS 500 mg: 51.2%, s |
| (41) | Randomized, PBO-controlled, double-blind, add-on  8-week baseline,  8-week titration,  12-week maintenance (ZNS 8 mg/kg/day),  3–4 -week taper or entry in LTFU | INCL: 6–17 years, uncontrolled FOS, 1–2 ASMs (no FBM or TPM), ≥4 FOS/month in 8-week baseline  EXCL: idiopathic generalized epilepsy, psychogenic seizures, cluster seizures, status epilepticus, progressive neurologic disease | *≥50% responder rate*:  PBO: 31%  ZNS: 50%, s  *Median % reduction in seizure frequency vs baseline*:  PBO: 24.5%  ZNS: 50.0%, s  *Seizure freedom*:  PBO: 3%  ZNS: 14%, s |

Add-on, adjunctive therapy; ASM, antiseizure medication; BRV, brivaracetam; CBZ(-CR), (controlled-release) carbamazepine; CNS, central nervous system; ESL, eslicarbazepine acetate; ETS, ethosuximide; EXCL, exclusion criteria; FBM, felbamate; FOS, focal-onset seizures; FU, follow-up; GBP, gabapentin; GOS, generalized-onset seizures; GTCS, generalized tonic-clonic seizures; INCL, inclusion criteria; LCM, lacosamide; LEV, levetiracetam; LSM, least square mean; LTFU, long-term follow-up study; LTG, lamotrigine; mono, monotherapy; ns, not statistically significant; OXC, oxcarbazepine; PBO, placebo; PER, perampanel; s, statistically significant; TGB, tiagabine; TPM, topiramate; VGB, vigabatrin; VPA, valproate; ZNS, zonisamide.

^a^Lists of inclusion/exclusion criteria and outcomes are not exhaustive.

**References**

1. Biton, V., Berkovic, S.F., Abou-Khalil, B., Sperling, M.R., Johnson, M.E., and Lu, S. (2014). Brivaracetam as adjunctive treatment for uncontrolled partial epilepsy in adults: a phase III randomized, double-blind, placebo-controlled trial. *Epilepsia* 55**,** 57-66. doi: 10.1111/epi.12433.

2. Ryvlin, P., Werhahn, K.J., Blaszczyk, B., Johnson, M.E., and Lu, S. (2014). Adjunctive brivaracetam in adults with uncontrolled focal epilepsy: results from a double-blind, randomized, placebo-controlled trial. *Epilepsia* 55**,** 47-56. doi: 10.1111/epi.12432.

3. Klein, P., Schiemann, J., Sperling, M.R., Whitesides, J., Liang, W., Stalvey, T., et al. (2015). A randomized, double-blind, placebo-controlled, multicenter, parallel-group study to evaluate the efficacy and safety of adjunctive brivaracetam in adult patients with uncontrolled partial-onset seizures. *Epilepsia* 56**,** 1890-1898. doi: 10.1111/epi.13212.

4. Elger, C., Halász, P., Maia, J., Almeida, L., and Soares-da-Silva, P. (2009). Efficacy and safety of eslicarbazepine acetate as adjunctive treatment in adults with refractory partial-onset seizures: a randomized, double-blind, placebo-controlled, parallel-group phase III study. *Epilepsia* 50**,** 454-463. doi: 10.1111/j.1528-1167.2008.01946.x.

5. Gil-Nagel, A., Lopes-Lima, J., Almeida, L., Maia, J., and Soares-da-Silva, P. (2009). Efficacy and safety of 800 and 1200 mg eslicarbazepine acetate as adjunctive treatment in adults with refractory partial-onset seizures. *Acta Neurol. Scand.* 120**,** 281-287. doi: 10.1111/j.1600-0404.2009.01218.x.

6. Ben-Menachem, E., Gabbai, A.A., Hufnagel, A., Maia, J., Almeida, L., and Soares-da-Silva, P. (2010). Eslicarbazepine acetate as adjunctive therapy in adult patients with partial epilepsy. *Epilepsy Res.* 89**,** 278-285. doi: 10.1016/j.eplepsyres.2010.01.014.

7. Sperling, M.R., Abou-Khalil, B., Harvey, J., Rogin, J.B., Biraben, A., Galimberti, C.A., et al. (2015). Eslicarbazepine acetate as adjunctive therapy in patients with uncontrolled partial-onset seizures: Results of a phase III, double-blind, randomized, placebo-controlled trial. *Epilepsia* 56**,** 244-253. doi: 10.1111/epi.12894.

8. Kirkham, F., Auvin, S., Moreira, J., Gama, H., Falcão, A.C., Rocha, J.F., et al. (2020). Efficacy and safety of eslicarbazepine acetate as adjunctive therapy for refractory focal-onset seizures in children: A double-blind, randomized, placebo-controlled, parallel-group, multicenter, phase-III clinical trial. *Epilepsy Behav.* 105**,** 106962. doi: 10.1016/j.yebeh.2020.106962.

9. Jóźwiak, S., Veggiotti, P., Moreira, J., Gama, H., Rocha, F., and Soares-da-Silva, P. (2018). Effects of adjunctive eslicarbazepine acetate on neurocognitive functioning in children with refractory focal-onset seizures. *Epilepsy Behav.* 81**,** 1-11. doi: 10.1016/j.yebeh.2018.01.029.

10. UK Gabapentin Study Group (1990). Gabapentin in partial epilepsy. *Lancet* 335**,** 1114-1117.

11. The US Gabapentin Study Group No. 5 (1993). Gabapentin as add-on therapy in refractory partial epilepsy: a double-blind, placebo-controlled, parallel-group study. *Neurology* 43**,** 2292-2298. doi: 10.1212/wnl.43.11.2292.

12. Anhut, H., Ashman, P., Feuerstein, T.J., Sauermann, W., Saunders, M., and Schmidt, B. (1994). Gabapentin (Neurontin) as add-on therapy in patients with partial seizures: a double-blind, placebo-controlled study. The International Gabapentin Study Group. *Epilepsia* 35**,** 795-801. doi: 10.1111/j.1528-1157.1994.tb02513.x.

13. Appleton, R., Fichtner, K., LaMoreaux, L., Alexander, J., Halsall, G., Murray, G., et al. (1999). Gabapentin as add-on therapy in children with refractory partial seizures: a 12-week, multicentre, double-blind, placebo-controlled study. Gabapentin Paediatric Study Group. *Epilepsia* 40**,** 1147-1154. doi: 10.1111/j.1528-1157.1999.tb00833.x.

14. Ben-Menachem, E., Biton, V., Jatuzis, D., Abou-Khalil, B., Doty, P., and Rudd, G.D. (2007). Efficacy and safety of oral lacosamide as adjunctive therapy in adults with partial-onset seizures. *Epilepsia* 48**,** 1308-1317. doi: 10.1111/j.1528-1167.2007.01188.x.

15. Halász, P., Kälviäinen, R., Mazurkiewicz-Beldzińska, M., Rosenow, F., Doty, P., Hebert, D., et al. (2009). Adjunctive lacosamide for partial-onset seizures: Efficacy and safety results from a randomized controlled trial. *Epilepsia* 50**,** 443-453. doi: 10.1111/j.1528-1167.2008.01951.x.

16. Chung, S., Sperling, M.R., Biton, V., Krauss, G., Hebert, D., Rudd, G.D., et al. (2010). Lacosamide as adjunctive therapy for partial-onset seizures: a randomized controlled trial. *Epilepsia* 51**,** 958-967. doi: 10.1111/j.1528-1167.2009.02496.x.

17. Farkas, V., Steinborn, B., Flamini, J.R., Zhang, Y., Yuen, N., Borghs, S., et al. (2019). Efficacy and tolerability of adjunctive lacosamide in pediatric patients with focal seizures. *Neurology* 93**,** e1212-e1226. doi: 10.1212/wnl.0000000000008126.

18. Baulac, M., Rosenow, F., Toledo, M., Terada, K., Li, T., De Backer, M., et al. (2017). Efficacy, safety, and tolerability of lacosamide monotherapy versus controlled-release carbamazepine in patients with newly diagnosed epilepsy: a phase 3, randomised, double-blind, non-inferiority trial. *Lancet Neurol.* 16**,** 43-54. doi: 10.1016/s1474-4422(16)30292-7.

19. Duchowny, M., Pellock, J.M., Graf, W.D., Billard, C., Gilman, J., Casale, E., et al. (1999). A placebo-controlled trial of lamotrigine add-on therapy for partial seizures in children. Lamictal Pediatric Partial Seizure Study Group. *Neurology* 53**,** 1724-1731. doi: 10.1212/wnl.53.8.1724.

20. Piña-Garza, J.E., Levisohn, P., Gucuyener, K., Mikati, M.A., Warnock, C.R., Conklin, H.S., et al. (2008). Adjunctive lamotrigine for partial seizures in patients aged 1 to 24 months. *Neurology* 70**,** 2099-2108. doi: 10.1212/01.wnl.0000285493.08622.35.

21. Glauser, T.A., Ayala, R., Elterman, R.D., Mitchell, W.G., Van Orman, C.B., Gauer, L.J., et al. (2006). Double-blind placebo-controlled trial of adjunctive levetiracetam in pediatric partial seizures. *Neurology* 66**,** 1654-1660. doi: 10.1212/01.wnl.0000217916.00225.3a.

22. Piña-Garza, J.E., Nordli, D.R., Jr., Rating, D., Yang, H., Schiemann-Delgado, J., and Duncan, B. (2009). Adjunctive levetiracetam in infants and young children with refractory partial-onset seizures. *Epilepsia* 50**,** 1141-1149. doi: 10.1111/j.1528-1167.2008.01981.x.

23. Brodie, M.J., Perucca, E., Ryvlin, P., Ben-Menachem, E., and Meencke, H.J. (2007). Comparison of levetiracetam and controlled-release carbamazepine in newly diagnosed epilepsy. *Neurology* 68**,** 402-408. doi: 10.1212/01.wnl.0000252941.50833.4a.

24. Barcs, G., Walker, E.B., Elger, C.E., Scaramelli, A., Stefan, H., Sturm, Y., et al. (2000). Oxcarbazepine placebo-controlled, dose-ranging trial in refractory partial epilepsy. *Epilepsia* 41**,** 1597-1607. doi: 10.1111/j.1499-1654.2000.001597.x.

25. Glauser, T.A., Nigro, M., Sachdeo, R., Pasteris, L.A., Weinstein, S., Abou-Khalil, B., et al. (2000). Adjunctive therapy with oxcarbazepine in children with partial seizures. The Oxcarbazepine Pediatric Study Group. *Neurology* 54**,** 2237-2244. doi: 10.1212/wnl.54.12.2237.

26. Piña-Garza, J.E., Espinoza, R., Nordli, D., Bennett, D.A., Spirito, S., Stites, T.E., et al. (2005). Oxcarbazepine adjunctive therapy in infants and young children with partial seizures. *Neurology* 65**,** 1370-1375. doi: 10.1212/01.wnl.0000186800.18456.72.

27. Schachter, S.C., Vazquez, B., Fisher, R.S., Laxer, K.D., Montouris, G.D., Combs-Cantrell, D.T., et al. (1999). Oxcarbazepine: double-blind, randomized, placebo-control, monotherapy trial for partial seizures. *Neurology* 52**,** 732-737. doi: 10.1212/wnl.52.4.732.

28. Sachdeo, R., Beydoun, A., Schachter, S., Vazquez, B., Schaul, N., Mesenbrink, P., et al. (2001). Oxcarbazepine (Trileptal) as monotherapy in patients with partial seizures. *Neurology* 57**,** 864-871. doi: 10.1212/wnl.57.5.864.

29. Beydoun, A., Sachdeo, R.C., Rosenfeld, W.E., Krauss, G.L., Sessler, N., Mesenbrink, P., et al. (2000). Oxcarbazepine monotherapy for partial-onset seizures: a multicenter, double-blind, clinical trial. *Neurology* 54**,** 2245-2251. doi: 10.1212/wnl.54.12.2245.

30. French, J.A., Krauss, G.L., Biton, V., Squillacote, D., Yang, H., Laurenza, A., et al. (2012). Adjunctive perampanel for refractory partial-onset seizures: randomized phase III study 304. *Neurology* 79**,** 589-596. doi: 10.1212/WNL.0b013e3182635735.

31. French, J.A., Krauss, G.L., Steinhoff, B.J., Squillacote, D., Yang, H., Kumar, D., et al. (2013). Evaluation of adjunctive perampanel in patients with refractory partial-onset seizures: results of randomized global phase III study 305. *Epilepsia* 54**,** 117-125. doi: 10.1111/j.1528-1167.2012.03638.x.

32. Krauss, G.L., Serratosa, J.M., Villanueva, V., Endziniene, M., Hong, Z., French, J., et al. (2012). Randomized phase III study 306: adjunctive perampanel for refractory partial-onset seizures. *Neurology* 78**,** 1408-1415. doi: 10.1212/WNL.0b013e318254473a.

33. Uthman, B.M., Rowan, A.J., Ahmann, P.A., Leppik, I.E., Schachter, S.C., Sommerville, K.W., et al. (1998). Tiagabine for complex partial seizures: a randomized, add-on, dose-response trial. *Arch. Neurol.* 55**,** 56-62. doi: 10.1001/archneur.55.1.56.

34. Sachdeo, R.C., Leroy, R.F., Krauss, G.L., Drake, M.E., Jr., Green, P.M., Leppik, I.E., et al. (1997). Tiagabine therapy for complex partial seizures. A dose-frequency study. The Tiagabine Study Group. *Arch. Neurol.* 54**,** 595-601. doi: 10.1001/archneur.1997.00550170069016.

35. Elterman, R.D., Glauser, T.A., Wyllie, E., Reife, R., Wu, S.C., and Pledger, G. (1999). A double-blind, randomized trial of topiramate as adjunctive therapy for partial-onset seizures in children. Topiramate YP Study Group. *Neurology* 52**,** 1338-1344. doi: 10.1212/wnl.52.7.1338.

36. Arroyo, S., Dodson, W.E., Privitera, M.D., Glauser, T.A., Naritoku, D.K., Dlugos, D.J., et al. (2005). Randomized dose-controlled study of topiramate as first-line therapy in epilepsy. *Acta Neurol. Scand.* 112**,** 214-222. doi: 10.1111/j.1600-0404.2005.00485.x.

37. Glauser, T.A., Dlugos, D.J., Dodson, W.E., Grinspan, A., Wang, S., and Wu, S.C. (2007). Topiramate monotherapy in newly diagnosed epilepsy in children and adolescents. *J. Child Neurol.* 22**,** 693-699. doi: 10.1177/0883073807303997.

38. Privitera, M.D., Brodie, M.J., Mattson, R.H., Chadwick, D.W., Neto, W., and Wang, S. (2003). Topiramate, carbamazepine and valproate monotherapy: double-blind comparison in newly diagnosed epilepsy. *Acta Neurol. Scand.* 107**,** 165-175. doi: 10.1034/j.1600-0404.2003.00093.x.

39. Wheless, J.W., Neto, W., and Wang, S. (2004). Topiramate, carbamazepine, and valproate monotherapy: double-blind comparison in children with newly diagnosed epilepsy. *J. Child Neurol.* 19**,** 135-141. doi: 10.1177/08830738040190020901.

40. Brodie, M.J., Duncan, R., Vespignani, H., Solyom, A., Bitenskyy, V., and Lucas, C. (2005). Dose-dependent safety and efficacy of zonisamide: a randomized, double-blind, placebo-controlled study in patients with refractory partial seizures. *Epilepsia* 46**,** 31-41. doi: 10.1111/j.0013-9580.2005.14704.x.

41. Guerrini, R., Rosati, A., Segieth, J., Pellacani, S., Bradshaw, K., and Giorgi, L. (2013). A randomized phase III trial of adjunctive zonisamide in pediatric patients with partial epilepsy. *Epilepsia* 54**,** 1473-1480. doi: 10.1111/epi.12233.
